# Factors related to help-seeking for cancer medical care among people living in rural areas: a scoping review

**DOI:** 10.1186/s12913-022-08205-w

**Published:** 2022-06-28

**Authors:** Mariko Oshiro, Midori Kamizato, Sayuri Jahana

**Affiliations:** Department of Nursing, Okinawa Prefectural College of Nursing, Yogi 1-24-1, Naha City, Okinawa, 902-8513 Japan

**Keywords:** Scoping review, Cancer, Help-seeking, Rural areas, Barriers, Facilitators, Ecological model of health behavior

## Abstract

Despite the importance of timely diagnosis and access to treatment, previous studies have not adequately explored help-seeking behavior in cancer treatment among rural and remote residents. The barriers preventing help-seeking behavior also remain unclear. To address this research gap, this study conducted a scoping review to suggest a framework for eliminating barriers and facilitating help-seeking for cancer treatment among rural and remote residents. To conduct the scoping review, three English medical databases (PubMed, MEDLINE, and CINAHL) were examined for the keywords “rural,” “remote,” “cancer,” and “help-seeking.” The research objectives and study designs, participants, and excerpts describing help-seeking of the selected papers were recorded in a data charting form. Descriptions of help-seeking behavior were organized and summarized according to their meaning and integrated into factors using thematic analysis. All extracted factors related to help-seeking were sorted into four main themes according to the Ecological Model of Health Behavior, the theoretical lens for this scoping review: (1) Intrapersonal; (2) Interpersonal; (3) Groups, culture, and organizations; and (4) Policy/environment. Factors were categorized as barriers and facilitators of help-seeking. A total of 13 papers were analyzed. Intrapersonal factors such as self-reliance, symptom appraisal, and fatalism, were identified as barriers to help-seeking, whereas presentation of abnormal and serious symptoms facilitated help-seeking. Interpersonal factors such as lack of understanding of family members, influence of surrounding people, role obligations, and lack of trust in experts hindered help-seeking, whereas understanding from surrounding people such as family and friends, promoted help-seeking. Groups, cultural, and organizational factors such as prejudice, social stigma, shame, lack of anonymity, and social norms acted as barriers to help-seeking. Policy-related barriers to help-seeking included lack of medical services and physical distance from medical institutions, leading to a time burden. The study discussed the identified factors from a rural context. Future studies should consider the identified barriers and facilitators according to the four main themes in rural areas when formulating interventions to promote help-seeking. Our findings can offer a theoretical foundation to develop actionable policies, preventive strategies, and relevant interventional tools that may facilitate oncological service utilization in rural areas.

**Supplementary Information:**

The online version contains supplementary material available at 10.1186/s12913-022-08205-w.

## Background

Previous studies have reported that there are differences between rural and urban areas in terms of timely access to healthcare [1], cancer survival rates [2], healthcare-seeking behaviors [3, 4], and financial problems [5]. In Japan, there are 405 designated regional cancer care hospitals to ensure that high-quality cancer treatment is available nationwide [6]. Despite this, cancer patients in Japan continue to face challenges in obtaining treatment, primarily due to the lack of accessibility and availability of designated cancer care hospitals in their areas of residence [7]. Healthcare professionals have reported a lack of access, psychological issues, and economic burdens related to help-seeking among cancer patients in rural areas [8].

The World Health Organization [9] mentioned that, to improve timely diagnosis and access to treatment, it is necessary to accurately understand current barriers in accessing care. This is necessary to design effective interventions to support early diagnosis and access to treatment [9]. Therefore, many existing reviews have focused on the help-seeking behavior of cancer patients to develop interventions for better cancer outcomes [10, 11]. According to Dobson [12], the path of help-seeking for cancer treatment among rural and remote residents is unclear. Moreover, studies focusing on help-seeking for cancer medical care among people living in rural areas are not well established. Existing new research agenda in 2020 advocated the need to clarify the help-seeking path of rural patients for cancer treatment and related factors based on their actual experiences to develop meaningful interventions to improve cancer outcomes for them [12]. Therefore, this study conducted a scoping review to provide a framework to identify ways to eliminate barriers to help-seeking for cancer treatment among rural and remote residents. This review had a specific and focused research question: What factors are associated with help-seeking for cancer treatment among rural residents in the literature? Our results can be used to develop actionable policies, preventive strategies, and relevant interventional tools that may help facilitate the use of oncological services in rural areas.

## Methods

### Study design

Our research objective was addressed using a scoping review, which is a type of knowledge synthesis approach used to map the concepts underpinning a research area and the main sources and types of evidence available [13–15].

#### Protocol and registration

Our protocol was undertaken using updated methodological guidance for conducting scoping reviews [16] and PRISMA -ScR guidelines [17].

#### Eligibility criteria

We set the inclusion criteria as follows: (1) published in a journal as an original paper, (2) written in English, (3) the study sample included adults living in rural and remote areas, and (4) focus on help-seeking for cancer treatment.

The term “rural” has been defined conventionally, subjectively, or geographically, and no definitive definition has yet been established; there is no single agreed definition yet [18–20]. In this study, all definitions of “rural” and “remote” used in the literature were accepted. Self-defined rural settings from any geographical region were included.

Further, based on previous studies of concept analysis for help-seeking behavior, we defined “help-seeking behavior” as a problem-focused, planned behavior for seeking medical help [21, 22].

We excluded the following studies: (1) samples with children, (2) evaluated interventions effects of related help-seeking, and (3) discussed special tests, such as genetic testing. The help-seeking for special tests may differ from those with more common help-seeking.

#### Information sources and search

Three English medical databases (PubMed, MEDLINE, and CINAHL) were searched using the keywords “rural,” “remote,” “cancer,” and “help-seeking.”

The search terms were refined using a four-step strategy. The strategy was developed not only for research teams but also with the advice from an informational researcher and a rural nursing researcher. Their inputs were useful in the refinement of key search terms which were most likely to produce the results sought. First, we considered related concepts such as “access to care,” synonymous words such as “seek help,” “seek,” and medical subject headings (MeSH) such as “neoplasms” (Table 1). These terms were extracted from related lectures by specialists, books, and relevant previous literature. Second, we searched (Table 1) databases (PubMed, MEDLINE, and CINAHL) using these specific words. Techniques for searching included the use of search tools such as subject headings and Boolean operators to narrow, widen, and combine literature searches (Additional file 1). Moreover, we searched for grey literature, including various sites such as Google. Third, we screened the search results, the titles, and abstracts, to assess whether the search terms reflect our research theme or not. Finally, we arrived at the specific search terms. After that, we confirmed whether the search terms used in this research had covered the main papers.

**Table 1:** The search terms for databases

RQ	Keyword	MeSH
Rural residents	rural, remote, frontier, snowfall, mountain, villages, islands	
Urban residents	metropolitan, urban, cities	cities
Help-seeking for cancer medical care	help-seeking, seek, seek help, access to care	
	cancer, malignant tumor	neoplasms

Papers published from 1991 to 2021 were included: 72 from PubMed, 37 from MEDLINE, and 37 from CINAHL. The search was conducted on July 30, 2021. The inclusion criteria were as follows: (1) published in a journal as an original paper, (2) written in English, (3) the study sample included adults living in rural areas, and (4) focus on help-seeking for cancer treatment. The exclusion criteria were as follows: (1) samples with children, (2) evaluated interventions effects of related help-seeking, and (3) discussed special tests, such as genetic testing.

#### Selection of sources of evidence

**Fig. 1:**
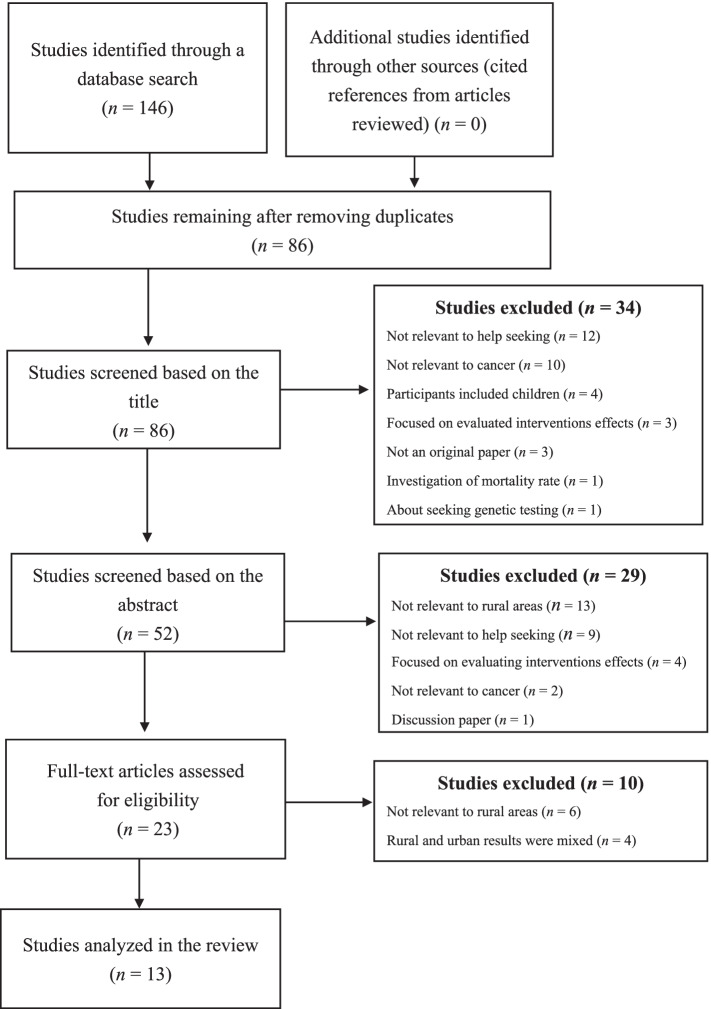
PRISMA flow diagram

A PRISMA flow diagram outlines the search and selection process [22] (Fig. 1). The title and abstract of each study were screened initially according to the inclusion and exclusion criteria. Papers selected at this stage were read in their entirety. Finally, eligible papers including factors associated with help-seeking by rural residents were analyzed in this review. Two reviewers (MO, MK) screened titles and abstracts for inclusion. Two reviewers (MO, MK) subsequently screened the full-text of potentially relevant articles to determine inclusion using similar inclusion and exclusion criteria. Subsequently, all included studies had been abstracted by the reviewers.

#### Data charting process

The research objectives and study designs, participants, and excerpts describing help-seeking behavior, were recorded in a data charting form (Table 2). Each included study was abstracted by the first reviewer (MO) and verified by the second reviewer (MK).

**Table 2:** Overview of studies that focused on help-seeking for cancer

Ref. No.	Author(s), year of publication	Country, region	Cancer type	Purpose	Study design	Targets; No. of samples	Excerpts of descriptions relating to help-seeking
[23]	Moodley et al., 2021	Uganda, South Africa (Africa)	Breast cancer, uterine cancer	To identify the factors that impede timely help-seeking behaviors when symptoms appear.	Quantitative	South Africa: 428 people in rural areas; 445 in cities Uganda: 427 people in rural areas; 458 in cities	In South Africa, those living in rural areas felt strongly that, compared with urban residents, they lacked the money to travel to and pay for medical institutions, and that their dialect and culture would not be understood by medical professionals. In Uganda, compared with urban residents, those living in rural areas were likely to have other role obligations and have husbands and/or partners who did not approve of help-seeking. Rural residents in both countries self-medicated when they became aware of cancer symptoms.
[24]	Goodwin et al., 2021	Australia	Various cancers	To identify awareness of delays when seeking help and factors related to the intention to seek help.	Quantitative	648 people residing in regional and rural areas who had been diagnosed with cancer	Being dismissive of problems, religion, the need for self-management, and having fatalistic views and attitudes were not associated with undergoing health screening examinations or delayed help-seeking.
[25]	Adsul et al., 2020	India (South Asia)	Uterine cancer	To identify the sociocultural factors that affect help-seeking for uterine cancer screening.	Qualitative	14 women residing in rural areas	Help-seeking was affected by shame and hesitation in discussing the uterus, a sex organ, as well as the belief that screening would not save them if it was their fate to develop cancer. Women had roles to play within the family and could not seek help without their husband’s permission. For some women who already had been diagnosed with cancer, their husbands and mothers-in-law remained unsupportive, and there was a stigma against cancer in the community. In addition to the financial burden of paying for the screenings themselves, women had to take time off work to undergo screenings.
[26]	Bergin et al., 2020	Australia	Colon cancer, Breast cancer	To compare the experience of patients living in rural and urban areas starting from receiving a diagnosis to undergoing treatment.	Qualitative	46 cancer patients residing in rural and urban areas	**Characteristics of rural residents:** In rural areas, it takes time to access the tests required to receive a diagnosis. In particular, there were no specialized hospitals in the area that could provide treatment, there were long waiting times to be attended to by specialists, and rural residents had other priorities, such as family and work. **Factors in common with those living in urban areas:** Help-seeking was delayed for the following reasons: did not think the symptoms were those of cancer; experienced similar symptoms in the past that were not due to cancer; felt that symptoms were natural and not problematic; in good health; the symptoms worsened gradually, so they were not linked to cancer; tried to manage cancer on their own; and felt that having cancer was shameful. Triggers that promoted help-seeking were as follows: development of abnormal symptoms and social recognition (e.g., being told by friends or other people around them that their symptoms might be cancer).
[27]	Goodwin et al., 2019	Australia	Colon cancer	To investigate the characteristics of patient attitudes and awareness related to help-seeking for colon cancer screening.	Quantitative	371 adults in the general population residing in rural, regional, and metropolitan areas	Compared with those living in regional and metropolitan areas, people in rural areas were significantly more dismissive of problems. This dismissiveness was associated with lower compliance with screenings and delayed help-seeking.
[28]	Steiness et al., 2018	Bangladesh (South Asia)	Breast cancer	To identify the factors that contribute to delayed help-seeking.	Qualitative	43 women residing in rural areas with breast cancer symptoms and 20 of their husbands	Participants identified having insufficient knowledge of breast cancer; inability to pay the costs of diagnosis and treatment; distant location of medical institutions; lack of doctors, laboratories, and pharmacies; lack of doctors whom they could trust; and having had an unpleasant past experience with a doctor. The following were also identified as barriers: fear of treatment and fatalism, views and attitudes toward disease (e.g., stigma), villagers’ cultural norms (e.g., not wanting to let women leave the village), and negligence or disinterest from family members (e.g., husbands and their family members did not allow their wives to receive the treatment). Religion was not associated with help-seeking.
[29]	Funnell et al., 2017	Australia	Skin cancer	To identify the factors that impede specialist help-seeking among patients receiving a skin cancer diagnosis.	Quantitative	201 adults in the general population residing in rural areas	Participants aged over 63 years and those who had lower education levels were more likely to solve problems on their own, be dismissive of problems, control their emotions so as not to let others see them, and distrust caregivers.
[30]	Mandengenda et al., 2014	Zimbabwe (Africa)	Various cancers	To identify the perceptions on cancer and the barriers to help-seeking as perceived by residents.	Quantitative	384 adults in the general population residing in rural areas	Lack of knowledge on cancer was cited as a barrier to help-seeking and was also associated with a low level of education.
[31]	Emery et al., 2013	Australia	Various cancers	To identify the factors associated with help-seeking during the interval between the presentation of symptoms and help-seeking.	Mixed-methods	66 adult cancer patients residing in rural areas	Factors associated with the delayed interval between becoming aware of cancer symptoms and seeking help were as follows: development of serious symptoms such as pain or dyspnea, geographic distance to medical institutions, optimism, stoicism, machismo, fear of undergoing medical tests, shame, and other role obligations.
[32]	Fort et al., 2011	Republic of Malawi (Africa)	Uterine cancer	To enhance the understanding of the barriers to help-seeking for uterine cancer screening.	Qualitative	20 women residing in rural areas	Participants’ knowledge on cancer screenings and symptoms was poor. Stigma against illness also influenced help-seeking. Additional barriers included a fatalistic view of cancer, securing time to undergo screenings and seek help, long waiting times at the hospital, and fear of whether they could afford the cost of screening. Facilitators of help-seeking included the appearance of symptoms, such as pain, and being able to receive support from neighbors, family members, and healthcare professionals.
[33]	Grunfeld et al., 2010	India (South Asia)	Breast cancer	To examine the beliefs about and help-seeking for breast cancer among urban- and rural-based Indian women.	Quantitative	Women in the general population (318 rural and 367 urban residents)	People residing in rural areas had poor knowledge of the symptoms of breast cancer and believed that cancer was a disease that always had a poor prognosis. They also tended to delay help-seeking even when they had become aware of the symptoms.
[34]	Schoenberg et al., 2010	USA	Uterine cancer	To identify the factors and circumstances surrounding a woman’s decision to seek follow-up treatment after receiving abnormal Papanicolaou test results.	Qualitative	27 women residing in Appalachia who had received abnormal Pap test results	Barriers to follow-up treatment including age < 18 or > 50 years; work, family, and other role obligations; the view that they could solve the problem on their own, lack of confidentiality owing to strong community ties, distrust of medical experts, not being accustomed to visiting the hospital because the family had never done so previously, fear, inability to pay treatment costs, lack of specialists, lack of community care, the time required to seek help or receive test results, dealing with different doctors during every visit, and insufficient means of transportation to reach the hospital with long waiting times.
[35]	Griffith et al., 2007	USA	Prostate cancer	To identify how the rural environment affects decision-making on treatment and screening	Qualitative	66 African-American men residing in rural areas	The participants cited the lack of medical services available to provide cancer treatment and the difficulty in obtaining information on treatment and screenings as barriers to help-seeking. Masculinity notions (e.g., a man would never seek treatment unless he felt pain and men should not seek medical help frequently) also influenced help-seeking. Participants also talked about feeling shame about having the disease (a disease of the male sex organ). They also discussed experiences, such as finding out that they had cancer after seeking help accompanied by another family member or having a family member who developed cancer and could not be saved, although they sought help at a hospital. These experiences suggest that social and family networks influenced help-seeking. Participants also did not see any benefits to undergoing cancer screening and receiving a diagnosis and said that being black and having a poor socioeconomic status affected help-seeking behavior.

#### Data items

We abstracted data on characteristics of the articles (e.g., type of article or study, country of corresponding author), population characteristics (e.g., type of cancer), and outcomes.

#### Synthesis of results

First, descriptions of help-seeking behaviors were organized and summarized according to their meaning and then integrated into factors using a thematic analysis. Discrepancies in thematic analysis were discussed among the study authors. Second, all extracted factors related to help-seeking from this study were sorted into “Factor of Barriers and Facilitators” (Table 3). Third, these Barriers and Facilitators factors were classified under subthemes in the column and mapped into four main themes in the ecological model of health behavior [36–39] (Table 3).

**Table 3:** Integration of factors related to help-seeking in rural areas

		Factor	Content
Barriers	Intrapersonal	Age	・Age > 63 years; dismissive of problems [29]
			・People aged < 18 or > 50 years refrain from help-seeking [34]
		Low educational level	・Low educational level [29, 30]
		Difficult financial conditions	・Financial burden [23, 25, 28, 32, 34, 35]
		Minority	・Feeling that their race, language, or culture will not be understood by doctors [23, 35]
		Fatalism	・Thinking that they will not survive if it is their fate to develop cancer [25, 28, 32]
		Self-reliance	・Self-medication [23]
			・Trying to control cancer by themselves [26, 29, 34]
			・Stoicism [31]
		Symptom appraisal	・Dismissive of problems/optimism [27, 29, 31]
			・Symptoms not linked to cancer [26]
		A lack of knowledge /awareness	・Inadequate awareness of cancer and its symptoms [28, 30, 32, 33]
			・Did not perceive any benefits of cancer screening or diagnosis [35]
		Fear	・Fear of tests and treatments [28, 31]
			・Fear of the financial burden of screenings and treatments [32, 34]
		Habits related to health services	・Not accustomed to visiting the hospital [34]
	Interpersonal	A lack of understanding from family members	・Cannot seek help without family members’ permission [23, 25, 28]
			・Family not supportive, even after developing cancer [25]
		Influence of surrounding people	・Having a family member who could not be saved [35]
			・Family with a long history of not availing medical services [34]
		Role obligations	・Roles in the family and other role obligations [23, 25, 26, 31, 34]
		Unreliable experts	・A lack of trust in doctors or caregivers [28, 29, 34]
			・Having had an unpleasant experience with a doctor [28]
	Groups/ cultures/ organizations	Prejudice/social stigma	・Community prejudice/social stigma against cancer [25, 28, 32]
		Shame	・Shame and timidity towards sex organs [25, 26, 31, 35]
		Lack of anonymity	・Lack of confidentiality due to strong community ties [34]
		Social norms	・Villagers not wanting to let women leave the village [28]
			・Machismo [31, 35]
	policy/ environment	Lack of medical services	・There is no specialized hospital in the area that can provide treatment [26, 28, 34, 35]
			・There are no doctors, laboratories, or pharmacies in their area of residence [28, 34]
			・There is no place to obtain information on treatment or screenings [35]
		Physical distance from medical institutions	・Medical institutions are located far away [28, 34]
			・Insufficient means of transportation to the hospital [34]
		Time burden	・It takes time to seek help and receive test results [26, 34]
			・Long waiting time to be attended to by specialists [26, 32]
Facilitators	Intra personal	Presentation of symptoms	・Presentation of symptoms such as pain [26, 31, 32]
	Inter personal	Understanding from surrounding people	・Support from neighbors, family members, and healthcare professionals [32]
			・Told by people around that it was cancer [26, 35]

Religion was not a factor in help-seeking [24, 29]

There was no association between help-seeking and fatalism, self-reliance and symptom appraisal [24]

The model considers rural and remote residents as individuals influenced by an ecosystem including political and other systems. Therefore, the ecological model was used as a theoretical framework in this study. This model conceptualizes the social world in four spheres or levels of influence. These levels of influence are: (1) Intrapersonal (individual characteristics that influence behavior such as knowledge, attitudes, beliefs, and personality traits); (2) Interpersonal [interpersonal processes and primary groups (family, peers, social networks, associations) that provide social identity and role definition]; (3) Groups, culture, and organizations (home environment/community organizations/informal structures such as religious groups, worksites, schools)]; (4) Policy/environment (healthcare policies/incentives/zoning codes/transportation, city planning) [36, 40].

## Results

A total of 13 papers were analyzed. Table 2 presents an overview of the papers included in this scoping review. All the selected papers were published after 2007. Five were from Australia, three from Africa, three from South Asia, and two from the US. Based on the study design, six were quantitative, six were qualitative, and one was a mixed-methods study. Table 3 shows the integration of factors associated with help-seeking in rural areas.

### Barriers

#### Intrapersonal

As shown in Table 3 [23–35], demographic factors such as *age* (age > 63 years, or aged < 18 or > 50 years) [29, 34], *low education levels* [29, 30], *difficult financial conditions* [23, 25, 28, 32, 34, 35], and *minority status* [23, 35] influenced help-seeking behavior. The papers from Africa [23, 32], US [34, 35], and South Asia [25, 28] reported that *difficult financial conditions* made people feel the burden of paying for travel to medical institutions for procedures like screening, diagnosis, and treatment.

An individual’s value such as *Fatalism* and *Self-reliance* influenced help-seeking behavior. The papers from South Asian and African countries, proven in the qualitative studies, reported *Fatalism* as one’s own fate to develop cancer [25, 28, 32]. Regarding *Self-reliance*, the paper from Africa reported the use of self-medication when they became aware of cancer symptoms [23]. The papers from Australia and US reported *self-reliance* such as trying to control cancer by themselves [26, 34], control their emotions so as not to let others see them [29], and stoicism and machismo [31].

*Symptom appraisal* such as being dismissive of problems/optimism and symptoms not linked to cancer were seen as barriers to help-seeking [26, 27, 29, 31]. The result of the quantitative study shows that, compared with those living in regional and metropolitan areas, people in rural areas were significantly more dismissive of problems [27]. For example, the people did not think the symptoms were those of cancer. Many experienced similar symptoms in the past that were not due to cancer and felt that they were natural and not problematic. They felt being in good health. As the symptoms worsened gradually, they were not linked to cancer [26].

The general *lack of knowledge/awareness* of cancer has emerged in several qualitative and quantitative studies as a relevant factor influencing help-seeking behavior [28, 30, 32, 33, 35]. The papers from South Asian [28, 33] and African [30, 32] countries reported inadequate awareness of cancer and its symptoms. For example, rural women believed that cancer was a disease that always had a poor prognosis [33]. A lack of knowledge regarding cancer was cited as a barrier in help-seeking and was also associated with a low level of education [30].

One study from every country reported the *fear* of tests and treatments and financial burden of screenings and treatments acting as barriers in help-seeking [28, 31, 32, 34].

*Health service utilization habits* are a barrier to help-seeking. For example, the people who are not accustomed to visiting the hospital because the family had never done so previously, tend to delay help-seeking [34].

#### Interpersonal

*Lack of understanding of family members*, was identified as a barrier to help-seeking in studies from South Asian and African countries [23, 25, 28]. Specifically, the person cannot seek help to receive treatment without family members such as husband’s and partner’s permission [23, 25, 28]. For example, for some women who already had been diagnosed with cancer, their husbands and mothers-in-law remained unsupportive [25].

In the US, the papers suggest that people were *influenced by help-seeking from their close circle* such as family members’ belief and experiences [34, 35]. For example, the family’s belief influenced hospital visits, as the family had never done so previously [34]. Another experience where a family member was diagnosed with cancer and could not be saved, despite seeking clinical assistance, could serve as a barrier to help-seeking [35].

*Role obligations* in the family, work and other priorities, are barriers to help-seeking in several countries such as Australia, the US, Africa, and South Asia [23, 25, 26, 31, 34].

*Unreliable experts* (e.g., the doctor did not listen carefully or displayed lack of cordiality) also act as barriers to help-seeking [28, 29, 34].

#### Groups, culture, and organizations

Community *prejudice/social stigma* against cancer affects help-seeking tendencies in Asian and African countries [25, 28, 32]. Further, help-seeking was affected by the *shame* of having cancer [26] and hesitation in discussing a sex organ such as the uterus [25, 35] in several countries, such as Australia, the US, and South Asia.

Additionally, *the lack of anonymity* and confidentiality create a burden on patients’ minds in close-knit populations like rural areas, which affects help-seeking behavior [34].

*Social norms* also affect help-seeking behavior. For example, there are villagers’ cultural norms (e.g., not wanting to let women leave the village) in South Asia [28]. Moreover, machismo (e.g., a man would never seek treatment unless he felt pain and men should not seek medical help frequently) also influenced help-seeking in Australia and the US [26, 30].

#### Policy/environment

The policy-related barriers to help-seeking were identified as *lack of medical services.* For example, absence of a specialized hospital in the area that can provide treatment [26, 28, 34, 35], lack of specialists such as doctors, laboratories or pharmacies in their area of residence [28, 34], and absence of a place to obtain information on treatment or screenings [35] contributed to this issue. This indicated that *physical distance from medical institutions* in rural areas hindered help-seeking. Medical institutions are located far away [28, 34], and insufficient means of transportation to the hospital [34] exacerbate the issue. Therefore, there is a *time burden* to access the tests required to receive a diagnosis and to seek help or receive test results. Additional factors include dealing with different doctors during every visit, and insufficient means of transportation to reach the hospital with long waiting times [26, 32, 34]. Help-seeking is associated with accessibility and availability of regional healthcare facilities and medical systems.

### Facilitators

#### Intrapersonal

*Presentation of symptoms* of abnormal conditions such as pain and other serious symptoms are facilitators of help-seeking [26, 31, 32].

#### Interpersonal

*Understanding from surrounding people* facilitates help-seeking [26, 32, 35]. Specifically, being told by people that it was cancer [26, 35], and receiving support from neighbors, family members, and healthcare professionals encourage help-seeking [32].

## Discussion

This scoping review explored factors associated with help-seeking for cancer treatment among rural and remote residents worldwide to develop actionable policies, preventive strategies, and relevant interventional tools that may help facilitate the use of oncological services in rural areas. The factors were grouped into four general categories based on the ecological model: intrapersonal, interpersonal, groups/cultures/organizations, and policy/environment. The diverse categories indicate that many varied factors impact help-seeking in rural settings.

### Principal findings and directions for future implication in rural areas

Our findings support factors such as age, low educational level, difficult financial conditions, symptom appraisal, lack of knowledge, fear, and habits related to health services, were associated with help-seeking. These intrapersonal factors are consistent with the components of a model depicting factors associated with help-seeking behaviors among patients with cancer [41]. In particular, the characteristics of help-seeking of cancer are symptoms experienced and so is symptom appraisal [42]. Symptom appraisal and presentation of symptoms have been reported in a majority of studies as barriers to help-seeking [26, 27, 29, 31] and facilitators of help-seeking [26, 31, 32]. When a person notices a bodily change or symptom, they perceive it a reason to seek medical help. Therefore, presentation of symptoms such as pain is one of the facilitators of help-seeking. Moreover, our review identified self-reliance (a “grin and bear it” attitude or trying to manage things on one’s own), and fatalistic views (the beliefs that one’s future health is predetermined by fate or destiny [43]) as barriers to help-seeking. In the rural areas, adversity was viewed as an inevitable part of life, and people were expected to cope with unexpected events as they occurred [44]. Rural people tend to accept ill-health with high degrees of stoicism and fatalism [45]. Thus, self-reliance and fatalism are viewed as values of rural people. When considering the directions for future intervention in rural areas, health service providers need to understand such rural characteristics and values when offering services.

In the interpersonal factors, our review revealed that factors such as a lack of understanding of family members, influence of surrounding people, role obligations, and a lack of trust in experts hindered help-seeking. In contrast, understanding one’s close circle, such as family and friends, promotes help-seeking. A nursing study conducted in rural areas reported that residents were closely connected and that family ties played a central role [37]. We recommend including the need for an array of studies and intervention approaches to advance help-seeking, to not only people in rural areas but also to their families using family-based approaches from these results.

In the group/cultural/organizational factors, our review also identified prejudice/social stigma, shame, lack of anonymity, and social norms as barriers to help-seeking. In rural areas, “small society” is still prevalent; thus, “everybody knows everybody” and the people’s daily lives are highly visible and open [44]. This scoping review shows that the regional cultural expectations such as lack of privacy and confidentiality in rural areas can constrain help-seeking, compounding a sense of isolation. This kind of rural context presents unique constraints in help-seeking, such as the lack of opportunities to consult feelings and experiences with others with similar personal, social, and cultural experiences. Hence, it is necessary to seek help inside and outside one’s community. According to a previous study that focused on rural areas, stigma has been related to a lack of knowledge, and educational interventions have been proven to be effective in reducing social prejudice and stigma in the community [46]. Therefore, educational interventions to spread awareness and knowledge about cancer may be effective in improving help-seeking among individuals in rural areas. Additionally, using technology-based communication, such as telehealth services, may enhance help-seeking for people living in rural areas [47].

Moreover, owing to the lack of medical resources in rural areas, residents often travel long distances to seek medical help. The resulting time burden is a barrier to help-seeking. Although these issues have been previously identified e.g., from policy-based perspectives [48], there remains a need for research to go beyond the help-seeking behaviors of individuals to investigate healthcare systems at the national level. Recently, evidence has shown that telehealth services can efficiently and effectively improve healthcare access and cost-effectiveness for rural and remote areas [47, 49]. We can consider retaining remote consultations alongside face-to face consultations in future routine healthcare services as this could improve access to healthcare in rural and remote areas.

### Implications for practice

This study extracted factors related to help-seeking for cancer medical care among people living in rural areas, including intrapersonal, interpersonal, groups/cultural/organizational, and policy/environmental factors. In order to develop actionable policies, preventive strategies, and relevant interventional tools, multi-level educational and health-promoting interventions should be implemented to reduce social stigma and to improve patients’ and their families’ understanding of cancer. Moreover, medical resources such as telemedicine should be set up and promoted.

### Limitations of the study

There are several limitations to our scoping review. First, we have used the unstandardized term for “rural” and “remote.” At present, some sections of the study are challenging in terms of establishing standardized terminology and national definitions of rural and urban areas [50]. Therefore, future studies should employ standardized terminology with “rural and remote” context using international statistics comparisons, such as degree of urbanization from new global agendas [50]. Second, our search was limited to only three databases and to studies published in English. This search based on the three databases might have led to omitting relevant articles. Thus, our results may not be generalizable. However, this review has highlighted many adequate comprehensive implications of rural and remote people’s help-seeking behavior for cancer medical care. The findings from the present review can be used as a starting point for future evidence-based strategies.

## Conclusions

The scoping review provides an overview of literature on the factors associated with help-seeking for cancer treatment among rural residents. This scoping review explored factors associated with help-seeking for cancer treatment among rural and remote residents worldwide to develop actionable policies, preventive strategies, and relevant interventional tools that may help facilitate the use of oncological services in rural areas. Factors related to help-seeking for cancer medical care can be categorized into four themes: intrapersonal, interpersonal, groups/cultures/organizations, and policy/environment using the ecological model. From the 13 selected articles, the barriers and facilitators were identified. These included understanding of people, self-reliance, fatalistic views, lack of anonymity, social norms, and lack of medical resources. Future studies should consider interventions to promote help-seeking, which must involve intrapersonal, interpersonal and rural community groups, culture, and organizations of each rural area.

## Supplementary Information

**Additional file 1.**

## Data Availability

All data generated or analyzed during this study are included in this published article.
