# Supplementary material for: Factors related to help-seeking for cancer medical care among people living in rural areas: a scoping review
Source: BMC Health Serv Res. 2022 Jun 28;22:836. doi: 10.1186/s12913-022-08205-w (PMC9241203; doi:10.1186/s12913-022-08205-w)
Supplement: Supplementary file 1 — Additional file 1. [file 12913_2022_8205_MOESM1_ESM.docx]

**Additional file 1. Search strategy**

PubMed

Updated 22/05/09 10:53

| S# | Search Terms | Search options | Results |
| --- | --- | --- | --- |
| 1 | ((rural, remote, frontier, snowfall, mountain, villages, islands) AND (cancer)) AND (help seeking) | **Search modes** - Find all my search  terms | 0 |
| 2 | ((rural) AND (help-seeking)) AND (cancer) | **Search modes** -  Find all my search terms | 35 |
| 3 | ((help-seeking, seek, seek help, access to care) AND (cancer, malignant tumor)) AND (rural, remote, frontier, snowfall, mountain, villages, islands) | **Search modes** -  Find all my search terms | 0 |
| 4 | (rural or remote) AND (help seeking) AND (cancer) | **Search modes** - Find all my search terms | 72 |
| 5 | ((rural, remote) AND (seek)) AND (malignant tumor) | **Search modes** -  Find all my search terms | 6 |
| 6 | ((access to care) AND (cancer)) AND (rural or remote) | **Search modes** - Find all my search  terms | 2006 |
| 7 | ((rural or remote) AND (seek help)) AND (malignant tumor) | **Search modes** -  Find all my search terms | 22 |
